# Factors influencing the natural regeneration of the pioneering shrub *Calligonum mongolicum* in sand dune stabilization plantations in arid deserts of northwest China

**DOI:** 10.1002/ece3.3913

**Published:** 2018-02-14

**Authors:** Baoli Fan, Allen David McHugh, Shujiang Guo, Quanlin Ma, Jianhui Zhang, Xiaojuan Zhang, Weixing Zhang, Juan Du, Qiushi Yu, Changming Zhao

**Affiliations:** ^1^ State Key Laboratory of Grassland Agro‐Ecosystems School of Life Sciences Lanzhou University Gansu China; ^2^ State Key Laboratory of Desertification and Aeolian Sand Disaster Combating Gansu Desert Control Research Institute Gansu China; ^3^ International Centre for Wheat and Maize Improvement (CIMMYT) Dhaka Bangladesh

**Keywords:** clonal propagation, pioneer species, seedling emergence, soil seed bank, soil type

## Abstract

*Calligonum mongolicum* is a successful pioneer shrub to combat desertification, which is widely used for vegetation restoration in the desert regions of northwest China. In order to reveal the limitations to natural regeneration of *C. mongolicum* by asexual and sexual reproduction, following the process of sand dune stabilization, we assessed clonal shoots, seedling emergence, soil seed bank density, and soil physical characteristics in mobile and stabilized sand dunes. Controlled field and pot experiments were also conducted to assess germination and seedling emergence in different dune soil types and seed burial depths. The population density of mature *C. mongolicum* was significantly different after sand dune stabilization. Juvenile density of *C. mongolicm* was much lower in stabilized sand dunes than mobile sand dune. There was no significant difference in soil seed bank density at three soil depths between mobile and stabilized sand dunes, while the emergence of seedlings in stabilized dunes was much lower than emergence in mobile dunes. There was no clonal propagation found in stabilized dunes, and very few *C. mongolicum* seedlings were established on stabilized sand dunes. Soil clay and silt content, air‐filled porosity, and soil surface compaction were significantly changed from mobile sand dune to stabilized dunes. Seedling emergence of *C. mongolicm* was highly dependent on soil physical condition. These results indicated that changes in soil physical condition limited clonal propagation and seedling emergence of *C. mongolicum* in stabilized sand dunes. Seed bank density was not a limiting factor; however, poor seedling establishment limited *C. mongolicum's* further natural regeneration in stabilized sand dunes. Therefore, clonal propagation may be the most important mode for population expansion in mobile sand dunes. As a pioneer species *C. mongolicum* is well adapted to propagate in mobile sand dune conditions, it appears unlikely to survive naturally in stabilized sand dune plantations.

## 1 INTRODUCTION

Pioneer species play an important ecological role in the stabilization of mobile sand dunes and the diversity of desert species. Pioneer species are hardy species, which are the first to colonize damaged ecosystems, beginning a chain of ecological succession that ultimately leads to a more biodiverse steady‐state ecosystem. As some uncolonized land may have thin, poor‐quality soils with few nutrients, pioneer species are often hardy plants with adaptations such as long roots, root nodes, and leaves that employ reduced transpiration strategies. Desert pioneer shrub species used in sand dune fixation in China, such as the *Calligonum mongolicum*, reduce wind erosion on the dune surfaces, enhance dust fall and deposition, facilitate colonization and the development of biological soil crusts (BSCs), as well as provide protection to soil crusts and herbaceous species (Gregory, Bruce, & William, 2001; Li, Kong, Tan, & Wang, 2007). However, if existing shrubs are unable to propagate naturally, sand dune reactivation is almost an irreversible process. Restoration studies indicate that populations of native sand pioneer shrub species decrease gradually, such as the annual *Agriophyllum squarrosum* and perennial *Artemisia halodendron* (Zuo et al., 2008), while herbaceous species that depend on increased soil nutrient levels gradually establish (Fan, Zhang, Ma, Li, & Zhao, 2016; Li, He, Duan, Xiao, & Jia, 2007; Wang, Li, Xiao, & Pan, 2006) and under natural regeneration, pioneer shrub species eventually disappear (Tobe, Zhang, & Omasa, 2005; Zhu, Dong, & Huang, 2009). Effective desertification control requires knowledge of natural regeneration processes of pioneer species in sand dunes. There is a considerable amount of literature on plant–environment relationships (Comstock & Ehleringer, 1992; Cook & Irwin, 1992; Monier & Wafaa, 2003; Parker, 1991; Yair & Danin, 1980; Zuo et al., 2008), but few discuss the mechanisms and/or occurrence of reduced natural regeneration of native species in post‐sand dune stabilization. Thus to fill this knowledge gap it is necessary to determine the biological and environmental limiting factors.

Generally, the viability of a soil seed bank and effective seedling emergence limits natural vegetation regeneration. On the one hand, seed banks are examined as a necessary first step for the design of ecological restoration plans (Baskin & Baskin, 1998; Boedeltje, Bakker, & TerHeerdt, 2003; Bossuyt & Honnay, 2008; Smith, McCormick, Leeds, & Garrett, 2002). Seed distribution in the seed bank is an important consideration in shaping the spatial distribution of seedlings (Bertiller, 1998; Kanowski, Catterall, Wardell‐Johnson, Proctor, & Reis, 2003); thus, differences in the soil seed bank density could result in differences in natural regeneration phenomena. On the other hand, for most plants, seed germination and seedling emergence represent critical life history stages, which are often subjected to high mortality (Harper, 1977). Knowledge of Seed germination and seedling establishment is critical in understanding the processes of species regeneration, especially for seedling emergence from complex soil (i.e., mobile sand), which is not just a matter of seed germination (Maun, 1994; Zheng et al., 2005) because soil texture can affect seed entrapment, burial, and seedling emergence (Chamber, 1995; Schneider, 2010). Worldwide studies in arid areas suggest that the stabilisation of mobile sand dune often accompanied with changes in topsoil properties, e.g. a continuous increase in silt and clay content coupling with the formation of BSCs and increasing vegetative covera”ge by herbaceous species (Burke, 2014; Li, He et al., 2007; Li, Kong et al., 2007; Su et al., 2007; Tavili & Jafari, 2009; Zhao, Guo, Zhou, & Drake, 2011). Additionally, the aboveground species composition, seed output, and longevity of individual seeds will influence the soil seed bank (Benvenuti, 2007; Coffin & Lauenroth, 1989). Thus, soil seed bank and seedling emergence between mobile and stabilized sand dune may vary due to changed environments. Understanding these changes would provide important insights into the natural regeneration dynamics of dune vegetation. However, much of our current knowledge of the processes of natural regeneration on sand dunes merely considers introduced species’ seed bank or seedling emergence. Few reports have integrated these elements in studies of population changes by natural regeneration (Kemp, 1989; Liu et al., 2011), and it remains relatively unknown whether changes in soil properties enhance or retard revegetation by natural regeneration in different restoration stages of sand dunes (Uselman, Snyder, Leger, & Duke, 2015). Assessing the success of a species for managed natural regeneration requires an integrated approach at a population level, which includes the whole of life cycle, because survival and regeneration of a species depend on adaptation to its changing habitat throughout the plant life cycle (Cao, Baskin, Baskin, Yang, & Huang, 2014).

*Calligonum mongolicum* (Turcz.) Bor. (Polygonaceae) is a native perennial xerophytic shrub, and a successful pioneer plant for desert vegetation succession. It has the widest geographic distribution of all *Calligonum* species (Shi, Pan, Gaskin, & Kang, 2009), and because of its sexual and asexual propagation strategies in sand dunes, it is widely used for vegetation restoration in the desert regions of northwest China. However, according to Tobe et al. (2005) and Zhu et al. (2009), and during our initial field observations on successional (stabilized) sand dunes, situated in the same region, population sparseness of plantation *C. mongolicum* was very evident, even though a mature shrub flowers profusely and can produce in excess of 120 seeds per shoot/stem, 2–3 times per year (unpublished data). Therefore, the existing mature *C. mongolicum* plantations used to combat mobile sand dunes provide an ideal research platform to study limitations in its propagation mechanisms under similar climatic conditions once dunes are stabilized. Thus, the aim of this paper was to determine limitations to population regeneration by either clonal or seed reproduction, following the process of sand dune stabilization. The selection of appropriate pioneer shrubs to stabilize sand dunes depends on its survival and procreation capacity until nonpioneer species can establish themselves. The impact of soil texture, soil seed bank density, and seedling emergence under natural populations planted across a number of decades and using pot experiments was considered in this study.

## 2 MATERIALS AND METHODS

### 2.1 Study site

The study was conducted in Minqin County (101°59′E–104°12′E, 38°08′N–39°26′N), on the lower reaches of Shiyang River at the eastern end of the Hexi Corridor, Gansu Province, northwest China. This area is largely surrounded by the Badain Jaran Desert in the northwest and the Tengger Desert in the east. The climate is arid desert with an average annual precipitation of 116.5 mm and an average temperature of 7.8°C and has 27.4 gale‐days per year at wind velocity ≥17 m/s with an annual mean wind speed of 2.4/ms. The soil at this site is classified as an Arenosol (SOTER_China, 2016), with part aeolian, part irrigated desert soils, and gray brown desert soil (OSU SCAS, 2004). Precipitation is usually the only source of water for desert plant growth (Du, Yan, & Dong, 2010). This site consisted of native xerophytic shrubs, small shrubs, and herbaceous plants; however, most trees and shrubs in this area are from plantation activities. The vegetative ground cover is generally <15% and this percentage cover generally is in decline. Mobile sand dunes are common in this desert landscape, which are sparsely populated with pioneer sand species such as *Agriophyllum squarrosum* and *C. mongolicum*.

### 2.2 Broader site characteristics

Following afforestation, mobile dunes near the oasis are generally stabilized through several years of effort (3 decades in this case), while mobile sand dunes outside of the oasis remain mobile due to the lack of large tree windbreaks and continuous sand movement greater than that in the desert to oasis transition zone. Once mobile sand dunes are stabilized with mature *C. mongolicum shrubs,* surface roughness is increased and soil surface conditions are obviously improved with biological soil crusts and some higher order herbaceous species and grasses such as *Chloris virgata, Setaria viridis, Salsola collina, Echinopilon divaricatum*, and *Eragrostis pilosa,* with the latter two species dominating the dunes.

### 2.3 Study species

*Calligonum mongolicum* is one of the major sand binding species grown in the sandy deserts of northwest China, which can propagate by asexual and sexual reproduction. Mature *C. mongolicum* are prolific producers of seeds, which have an elliptical achene with a very hard pericarp, and therefore, general predation or consumption and dispersal by herbivores are not common. Fortunately, the prevailing wind disperses seed over considerable distances, where on deposition the mobile sand buries them to various depths, but even so, after emergence and establishment, *C. mongolicum* tends to be sparsely populated on mobile and semimobile sand dunes.

### 2.4 Sampling the *C. mongolicum* inventory

Toward the end of the active growing season of a 30‐year‐old mature plantation in September 2013, the density of mature and juvenile (<30 cm in height) *C. mongolicum* was assessed in six 20 × 20 m^2^ plots on the windward sides of mobile and stabilized dunes. During sampling, to determine whether mature or juvenile plants came from sexual (Seed) or asexual (clonal) reproduction, roots were excavating to assess if the plants had developed from vegetative nodules (ramets) on horizontal roots. These ramets were counted in clusters on the horizontal roots. Additionally, the number of mature mother plants, the number of dead shoots on the shrubs for each shrub, seedling type, and seedling emergence (1‐year‐old seedling) in each sample were also recorded. The population density, seed seedling density, and clonal seedling density were determined from the number of mature shrubs and seedling type divided by the plot area, respectively.

### 2.5 Soil sampling measurements

Three 400‐m^2^ (20 × 20 m) plots at least 100 m apart were selected randomly on the windward sides of each mobile and stabilized sand dune sites with similar slopes and exposure within a 30‐year‐old mature plantation. The windward side of mobile sand dune sites consisted of buried and eroded sand surfaces, while the ground surface of the stabilized sand dunes was almost 65% crusted and covered (12.4%) with herbaceous plants. Within each *C. mongolicum* plot, three sampling ditches were dug, from where five soil samples were collected manually by mixing five subsamples collected from each soil layer at two soil depths of 0–2 cm and 2–10 cm. Stainless steel cylinders (100 cm^3^ in volume) were used to sample soil to determine bulk density and water content. The soil water content at each depth was determined gravimetrically on an oven‐dry mass basis after drying samples at 105°C for 48 hr. Soil compaction was measured from depths of 0–10 cm by a soil compaction tester (SC 900, USA). Air‐filled porosity was calculated from soil bulk density and empirical absolute particle density (2,650 kg/m^3^) after Liang, Zhang, and Wong (1996). Additional soil samples were collected to determine particle size distribution (PSD). Samples were air‐dried in the laboratory, after which gravel and roots in the samples were carefully removed. PSD was determined by laser diffraction equipment (Mastersizer 2000, UK) and divided into four particle sizes: clay content (<0.002 mm), silt content (0.002–0.02 mm), fine sand (0.02–0.2 mm), and coarse sand (0.2–2 mm).

### 2.6 Soil seed bank

To understand the distribution pattern of buried seed and its effects on seedling emergence in mobile and stabilized sand dune plots, soil seed bank samples were collected prior to the active growing season (in early May 2013). In each 20 × 20 m plot, five sites were randomly selected, in which a 20 × 20 cm square canisters were used to sample the soil at three depths (0–2, 2–5, and 5–10 cm). *C. mongolicum* seeds are generally 3.9 ± 0.2 mm in diameter and were separated by sieving samples through a 1.0‐mm sieve. To determine seed viability, a subsample of seed was debristled and soaked in a 1% solution of 2, 3, 5‐triphenyl‐2H‐tetrazolium chloride (TTC) for 48 hr to determine seed hardness and embryo color (Baskin & Baskin, 1998). Hard seeds with light‐colored embryos were recorded as viable, while soft seeds with dark embryos were recorded as nonviable.

### 2.7 Seed collection

Over the previous three, and the current active growing seasons, fresh seeds from *C. mongolicum* were collected from the surface of desert sand dunes in Minqin County. These were air‐dried in paper bags and stored in year‐collected batches for use in germination and emergence pot trials. Mean seed weight of the collected *C. mongolicum* seeds was 0.11 g ± 0.00 with a length of 1.64 ± 0.16 cm. Bristle length, which aids dispersal, was 4.15 ± 0.91 mm at around 25 percent of the length of the seed.

### 2.8 Controlled field and pot experiments

Concurrent pot experiments were conducted to assess emergence responses to soil type, and burial depth. Mobile sand dunes generally consist of a sandy soil, while stabilized sand dune surfaces are more consistent with a sandy loam soil. Thus, for the seed burial assessment, sandy soil and sandy loam soil were taken from mobile and stabilized dunes nearby the plantation plots in this study. Surface soil from each dune type, to a depth of approximately 7 cm, was collected and mixed to ensure homogeneity in each treatment pot. Strips of nylon mesh were placed over the drainage outlets to prevent soil loss, while allowing drainage of any excess water. Soil layering and tapping of the pot on a hard surface ensured that pot soil bulk density was similar to field conditions. The experiment consisted of five replicates of 20 seeds per burial depth for each soil type. Seeds that were dry‐stored for 1 year were manually placed on the soil surface in plastic pots and then covered by soil at burial depths of 0, 1, 2, 5, and 7 cm. The potted sand dune soil dried quickly in the hot, dry conditions of our study site; therefore, the pots were moistened daily from a garden watering can fitted with a very fine rosette to simulate light rain, the volume of which, was based on previous unpublished work that had indicated that flooding‐type irrigation limited *C. mongolicum*'s emergence. Therefore, we mimicked light rainfall in very small quantities, as would be the case in the desert, that is, moistened soil with maximized air‐filled porosity. Meanwhile, seedling appearance at the sand surface was recorded daily for 3 months, and on completion of the study. The percentage of seedling emergence was the number of seedlings divided by the number sown.

### 2.9 Survival rate of seedling emergence

Seedling survival rate and emergence in the field were assessed by sowing 50 seeds in 20 × 20 cm^2^ plots in early spring. Each plot was replicated 5 times in three sand dunes of each sand dune type. Seedling emergence was assessed in June, and seedling survival was assessed at the end of September. The seedling survival rate was determined from the number of survival seedlings divided by the number of emerged seedlings.

### 2.10 Statistical analysis

The statistical package SPSS 16.0 software (SPSS, Chicago, IL, USA) was used for the analysis. An ANOVA was used to analyze the difference in population regeneration states, plant population status, and soil physical conditions in mobile and stabilized sand dunes. A two‐way ANOVA was used to analyze the effect of sand burial, soil type, and sand burial interaction on seedling emergence. Where there was a significant difference, a multiple comparison LSD test was used to determine the level of difference among treatments at *p* < .05. All data on seedling emergence and seed bank density were arcsine‐transformed before statistical analysis in order to ensure homogeneity of variance. Data means ± *SE* and figures were created with Origin 8.0.

## 3 RESULTS

### 3.1 Population regeneration and population states of *C. mongolicum*

Natural regeneration of *C. mongolicum* was markedly different on stabilized and mobile sand dunes. Seedlings from sexual and asexual reproduction occurred on mobile sand dunes; however, the latter was the dominant mode, at 23.8 times greater than seedlings from seed. Clonal propagation did not occur on the stabilized dune sites; however, there were small number of seedlings from seed, which was 21% of the number occurring in mobile sand dunes (Table 1).

**Table 1:** Population regeneration states of *C. mongolicum* at stabilized and mobile sand dunes

	Seed seedling density (number/ha)	Clonal seedling density (number/ha)
Stabilized sand dune	67.0 ± 33.0	0
Mobile sand dune	313.0 ± 65.0	7,440 ± 2,570
*F*	9.40	4.72
Sig.	*P *< .05	*P *< .05

After 30 years of growth, shrubs in the plots in the mobile sand dunes were largely eroded by wind, while in stabilized sand dune shrubs experienced slight sand deposition. The density of mature *C. mongolicum* shrubs on mobile sand dunes was 3 times the number per m^2^ of those on stabilized sand dunes, which were also significantly taller, with a higher number of leaves per shoot. Meanwhile, the percentage of dead shoots was significantly more in stabilized sand dunes compared with mobile sand dunes (Table 2).

**Table 2:** Population states of *C. mongolicum* in stabilized and mobile sand dunes

	Stabilized sand dune	Mobile sand dune
Burial/Erosion depth (cm)	5.68 ± 0.392	15.04 ± 1.56
Population density (*N*/m^2^)	0.117 ± 0.003b	0.348 ± 0.077a
Dead shoots percentage (%)	62.04 ± 4.04a	38.48 ± 7.77b
Height (cm)	96.6 ± 12.3b	133 ± 9.5a
Ground diameter (cm)	23.63 ± 4.58a	26.83 ± 5.91a
Crown area (m^2^)	1.01 ± 0.19a	1.32 ± 0.07a
Leaf number/cm shoot	0.44 ± 0.03b	2.68 ± 0.66a

Different lowercase letters after each population state indicate significant differences (*p* < .05) between mobile sand dune and stabilized sand dune.

### 3.2 Soil physical properties of mobile and stabilized sand dunes

According to the PSD data, mobile sand dunes contained very limited clay and silt fractions, nearing 0%. Percentage of course and fine sand tended to approach 50% at each depth. In stabilized dunes, clay (~2%) and silt (7%) in the soil surface (0–2 cm) were significantly different to that at lower depths (2–10 cm) at 0.21 and 2.9%, respectively (Table 3).

**Table 3:** Descriptive soil properties of stabilized and mobile sand dunes in Minqin County

Soil Properties	Mobile sand dune		Stabilized sand dune	
Soil depth	0–2 cm	2–10 cm	0–2 cm	2–10 cm
Clay	0^Ab^	0^Ab^	1.90 ± 0.76^Aa^	0.21 ± 0.09^Ba^
Silt	0^Ab^	0^Ab^	6.79 ± 2.18^Aa^	2.88 ± 0.54^Ba^
Fine sand	48.88 ± 5.13^Aa^	49.65 ± 7.79^Aa^	51.01 ± 7.19^Aa^	45.42 ± 3.56^Aa^
Coarse sand	51.12 ± 5.13^Aa^	48.88 ± 7.80^Ab^	40.31 ± 8.76^Ab^	51.49 ± 3.23^Aa^
Soil Water content (%)	1.40 ± 0.43^Aa^	2.07 ± 0.67^Aa^	1.49 ± 0.42^Aa^	1.54 ± 0.49^Aa^
Bulk density (g/cm^3^)	1.57 ± 0.03^Aa^	1.61 ± 0.01^Aa^	1.52 ± 0.01^Aa^	1.55 ± 0.03^Aa^
Air‐filled porosity (%)	19.60 ± 1.8^Aa^	18.10 ± 1.5^Aa^	11.21 ± 1.5^Bb^	15.50 ± 1.61^Aa^

Different uppercase letters indicate significant differences (*p *< .05) in soil properties between two soil depths in the same sand dune site. Different lowercase letters indicate significant differences (*p *< .05) in soil properties between two sand dune types at the same soil depth.

**Figure 1:**
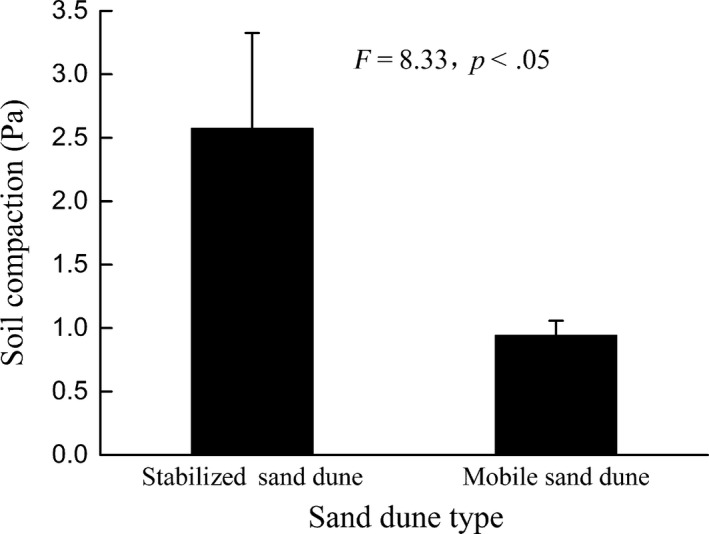
Soil compaction of stabilized and mobile sand dunes in plots of *Calligonum Mongolicum*

Coarse sand percentage at the soil surface of stabilized dunes was significantly reduced (40%) in comparison with that of mobile dunes (51%). Although bulk density was not significantly different across dune types, air‐filled porosity was significantly less at 11.2% in the surface of stabilized dunes compared to 19.6% in mobile dunes (Table 3). Meanwhile, soil surface compaction in stabilized sand dunes was significantly 2.7 times greater than that in the mobile sand dunes (Figure 1).

### 3.3 Seed distribution patterns in soil seed bank

**Figure 2:**
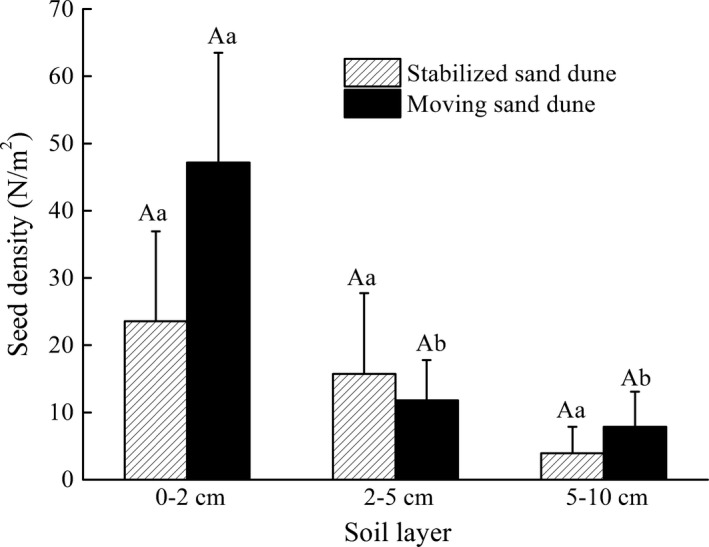
Seed density of mature *Calligonum mongolicum* seed bank at three soil depths on mobile and stabilized sand dunes in Minqin County. (Different uppercase letters indicate significant differences (*p* < .05) in soil seed bank density between two sand dune types at the same soil depth. Different lowercase letters indicate significant differences (*p* < .05) in soil seed bank density among different soil depths within sand dune types)

Soil depth, sand dune type, and their interactions had no significant effect on soil seed bank density (Table 4). There was no significant difference in seed distribution between dune types. Seed density near the surface of mobile sand dunes was significantly larger than other depths, but there was no difference in seed density between depths in stabilized sand dunes (Figure 2).

**Table 4:** Two‐way ANOVA of the effect of soil depth, dune type, and their interactions on soil seed bank density

Source	SS	*df*	MS	*F‐*value	*p‐*Value
Soil depth	1904.615	2	952.308	1.22	.303
Dune type	231.642	1	231.642	0.297	.588
Soil depth × Dune type	463.285	2	231.642	0.297	.744
Error	42158.92	54	780.721		

### 3.4 Pot trials on effects of soil type and sand burial depth on seedling emergence

**Figure 3:**
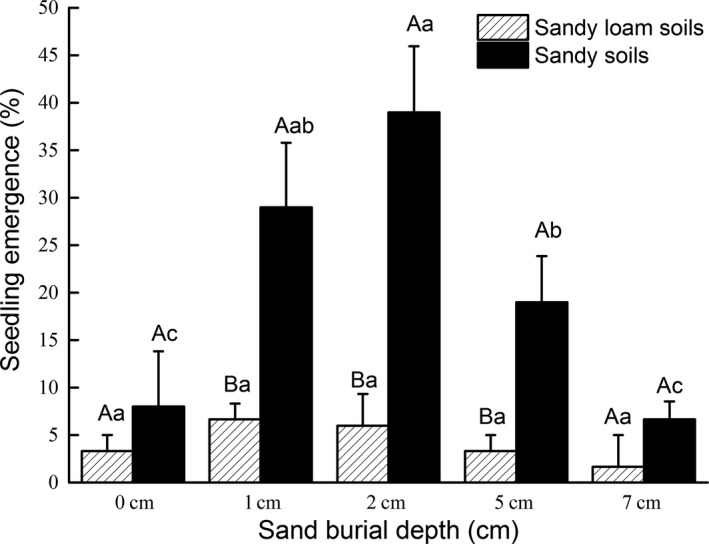
*Calligonum mongolicum* seedling percentage emergence (mean ± *SE*) on two soil types (sandy and sandy loam) at seed burial depths of 0, 1, 2, 5, 7 cm, after 30 days’ incubation. (Different uppercase letters indicate significant differences (*p* < .05) in seedling emergence between the two soil types at the same burial depth. Different lowercase letters indicate significant differences (*p* < .05) in seedling emergence among different sand burial depths within the same soil type)

Soil type had a significant effect on seedling emergence in pot trials (Table 5), where emergence in sandy soil from mobile dunes ranged from <10% to 40% across all depths. However, emergence in sandy loam soil from stabilized dunes was <6.7% across all depths. Seed burial depth had a significant effect on emergence in sandy soils, but it was not a significant factor in sandy loam soils. At 2 cm seed burial depth in sandy soils, emergence was 40% and significantly larger than 0, 5, and 7 cm burial depths. Emergence from 1 cm depths was 29%, which was considerably improved on seeds buried at 0 and 7 cm, but not significantly different from seed buried at 2 and 5 cm at 40% and 20% emergence, respectively (Figure 3).

**Table 5:** Two‐way analysis of variance on the effects of soil type, sand burial depth, and their interactions on seedling emergence of *Calligonum mongolicum*

Source of variance	SS	*df*	MS	*F*‐value	*p*‐Value
Soil type	97.177	1	97.177	9.468	<.05
Sand burial depth	417.94	4	104.485	10.18	<.0001
Soil type × Sand burial depth	216.776	4	54.194	5.28	.05
Error	205.267	20	10.263		

### 3.5 Seedling emergence and survival in sand dunes

Percentage seedling emergence from the mobile sand dune was eight times higher than stabilized sand dunes (Table 6). At the end of growing season, seedlings that emerged in spring did not survive in stabilized sand dunes. Meanwhile in mobile sand dunes, very few emerged seedlings survived (Table 6).

**Table 6:** Effects of sand dune type on the seedling emergence and survival rate of *Calligonum mongolicum*

	Seedling emergence percentage	Seedling survival percentage
Mobile sand dune	8.0 ± 2.55	1 ± 1
Stabilized sand dune	1.0 ± 0.67	0
Mean square	163.333	3.333
*F*	12.49	2.167
Sig.	0.004	0.165

## 4 DISCUSSION

The stabilized sand dunes sampled in this study where mobile sand dunes about thirty years ago when *C. mongolicum* and other desert shrubs were first planting. Unfortunately, as the dunes stabilized over the last 3 decades, anecdotal field observations indicated that natural regeneration of *C. mongolicum* was very poor, compared with populations on mobile sand dunes. This present study indicated that *C. mongolicum* was not able to increase its population by vegetative propagation and was largely dependent on seedling establishment. However, very few *C. mongolicum* seedlings established themselves on stabilized sand dunes. Consequently, mature population density on stabilized sand dunes was considerably less than that on mobile sand dunes (Table 3). This could be attributed to significantly larger percentage of dead shoots on mature plants in stabilized sand dunes compared with mobile sand dunes. The incidence of increased dead shoots in stabilized sand dune corresponds with a number of previous studies. These describe the communities being dominated by planted shrubs during mobile sand dune stabilization, which are gradually replaced by shallow‐rooted herbaceous species due to changes in the soil moisture and BSCs (Fan et al., 2016; Li et al., 2010; Wang et al., 2006). The deeper rooted shrubs, especially in older vegetation areas (stabilized dunes), critically modify the vertical distribution of soil water content, which is much lower than in relatively younger vegetation areas (mobile dunes; Li, Kong et al., 2007); thus, after soil water is depleted, shrub mortality begins to occur.

### 4.1 Soil physical limitation for clonal propagation

Clonal propagation of the *C. mongolicum* was not found in the stabilized sand dunes of our research plots. One possible explanation is that ramets in stabilized sand dunes were too deeply buried by sand and unable to break through to the surface. This result is consistent with studies on *Calligonum arborescens*, where particular sand burial depth was an essential prerequisite for rhizomes to survive (Luo & Zhao, 2015). Deep sand burial could limit gaseous exchange and, in the absence of light, reduce elongation and subsequent leaf formation (Luo & Zhao, 2015; Samsone et al., 2009). In the present study, soil air‐filled porosity was reduced, with increased soil compaction in stabilized sand dunes (Table 2 and Figure 2), which may have retarded the ramets development, emergence, and survival (Maun, 1994).

### 4.2 Limitation in regeneration by seed

#### 4.2.1 Soil seed bank

According to Moody‐Weis and Alexander (2007), seed survival and germination are functions of seed depth in the seed bank. The vertical seed bank distribution is of fundamental importance because seedling emergence either decreases continuously with seed depth or increases with slight burial and then decreases at greater depth (Colbach, Roger‐Estrade, Chauvel, & Caneill, 2000). Sand burial depth changes the soil environment for seed germination; thus, germination of seeds is directly related to the depth at which seeds are buried (Baskin & Baskin, 1989; Benvenuti, Macchia, & Miele, 2001; Gutterman, 1993; Liu et al., 2011; Zhang & Maun, 1990; Zhu et al., 2009, 2014). In our pot experiment, seedling emergence was significantly affected by sand burial depth, during which we determined the optimum depth for seedling emergence was 2 cm, which indicated that subsequent population regeneration was dependent on not only seed bank density, but also the vertical distribution pattern in the seed bank (Liu et al., 2011). As reported by Ma and Liu (2008), seed bank density in different microsites affected seedling emergence of *Agriophyllum squarrosum* (Chenopodiaceae) in northeastern Inner Mongolia. Our results showed that the density of soil seed bank in the moving sand dunes is higher than that of fixed dunes due to a significantly larger percentage of dead shoots, which pointed to a potential loss of fruit in stabilized sand dunes than in mobile sand dunes, although the vertical distribution of seed bank density between mobile and stabilized sand dunes was not significantly different. Thus, seed bank density between sand dune types was not a limiting factor for natural regeneration of *C. mongolicum* in this case. Additionally, seed density on the surface of moving sand dunes was significantly larger than other depths (Figure 1), which could be due to sampling on the windward side, where sand erosion may have expose more seeds reducing chances of burial; thus, fewer seeds were found in deeper soil.

The composition of the seed bank is influenced by aboveground species composition, seed output, and longevity of individual seeds (Benvenuti, 2007; Coffin & Lauenroth, 1989). The expectation would be that once the sand surface was stabilized with shrubs, grasses, other herbaceous plants, litter, or depressions, the windblown *C. mongolicum* seeds could be more easily captured for soil burial. However, the shrubs are prolific producers of flowers and seed, but it would appear that equivalent quantities of seed were not reflected in the seed bank in both sand‐type dunes. *C. mongolicum* fruit has seed bristles that aid wind dispersal, which, even at low wind speed, they can be carried considerable distances, and will generally only land when encountering an obstacle. An important feature of mobile sand dune habitats compared with stabilized sand dunes is the stronger wind erosion and reduced ground coverage; therefore, neither dune types tend to favor seed capture and establishment as evidenced by the low number of seeds reported in the seed bank. Therefore, we largely attributed the disparity between potential seed produced and soil seed bank, to wind dispersal and limited capture opportunities.

#### 4.2.2 Soil limitations for seedling emergence and propagation

In our study, seedling emergence in sandy loam soils (stabilized dunes) was much lower at moderate depths (1–5 cm) than in sandy soil (mobile dunes), while numerous previous studies have reported that soil type is an important factor in seed germination and seedling emergence (Melander & Kristensen, 2010; Rayamajhi, Pratt, Tipping, & Center, 2012; Uselman et al., 2015). Various stresses or conditions related to soil texture could restrict seed germination and seedling emergence, which affect further pioneering, and subsequently control the distribution and composition of vegetation in dune ecosystems (Dech & Maun, 2005; Hesp, 1991). According to Li, He et al. (2007), Li, Kong et al. (2007), the soil condition is altered during the process of mobile sand dune stabilization, affecting pioneering establishment and succession. In the current study, surface soil of stabilized sand dunes had significant increase in clay and silt content and lower porosity compared with mobile dunes. Air‐filled porosity is a sensitive measure, reflecting the ability of the seedling roots to forage and grow (Yang, Zhang, Zhao, Ruan, & He, 2005), and it is critical for normal aerobic soil activity (i.e., root and microbial respiration), as gas diffusion increases exponentially as air‐filled porosity increases (Freijer, 1994). Several studies have shown that root growth could be severely impeded when the air‐filled porosity falls below 10% of the total soil volume, largely due to poor gaseous exchange (Childs, Shade, Miles, Shepard, & Froehlich, 1989; Theodorou, Cameron, & Bowen, 1991). Our results demonstrated that seedling emergence of *C. mongolicum* responded differently between sand dune types. Stabilized sand dune soils had lower air‐filled porosity (11%) at the same depth compared to mobile sand dune, which confirms that air‐filled porosity may have played a role in limiting seedling growth. Additionally, seedling emergence may also have been affected by surface crusting (mechanical impedance) caused by an increase in other soil particle fractions and formation of biological crusts. Seedling establishment is dependent upon root growth and shoot elongation for the physical force needed for emergence. The perceived improvement in soil properties under sand dune stabilization did not necessarily create a favorable environment for seed germination and seedling emergence of *C. mongolicum,* which is largely a pioneering species in mobile sand dunes in which seedlings can emerge and grow more easily in the sandy conditions. It would appear that *C. mongolicum* seedling recruitment and propagation in sand dunes are intimately tied to the physical conditions of mobile sand to bury the seed and promote conditions that break dormancy and seedling propagation. This study clarified the points made by Kemp (1989) that changes in soil properties brought about by pioneering plants do indeed, at some point during decadal restoration of sand dunes, retard propagation of pioneering species, allowing for succession of nonpioneering species.

Emerged seedlings represents the most sensitive part of the plant life cycle (Harper, 1977), and seedlings are commonly subject to high mortality rates (Silvertown & Charlesworth, 2001). Seedling survival of *C. mongolicum* in mobile and stabilized sand dunes was very low (Table 6), indicating that the seedlings were very vulnerable in the desert environment, which appeared to restrict natural regeneration in the dunes. In stabilized sand dunes, there was an abundance of herbaceous plants and biological crusts resulting in less soil water content at depth than mobile sand dunes, thus limiting seedling survival once soil surface layer moisture was exhausted.

*Calligonum Mongolicum* has evolved as a pioneer species, and under the extreme arid mobile sand dune conditions of Minchin County, it favors to reproduce asexually in order to increase reproductive success, locally. However, as a pioneering species it will eventually decline, as indicated in this study, due to changed dune conditions, from clay and silt deposition, by creating plant litter that decomposes and creating new soil and nutrients for secondary succession.

## 5 CONCLUSIONS

Natural regeneration by the shrub *C. mongolicum* in stabilized sand dunes is potentially limited by the absence of clonal regeneration, low seedling emergence, and incidence of dead shoots in mature plants. Reduced air‐filled porosity and increased soil compaction in stabilized sand dunes retarded ramet development, and thus, clonal propagation was not found in stabilized sand dunes. For sexual reproduction, the soil seed bank density was not a limiting factor in natural regeneration in stabilized sand dunes. Seedling emergence was optimized at a seed burial depth of 2 cm; however, changed soil physical conditions related to air‐filled porosity, compaction, and surface crusting retarded seedling emergence and establishment by sexual reproduction in stabilized sand dunes. Overall seedling survival in mobile and stabilized dunes was relatively low, which suggests that at particular stages of seedling survival and seedling growth could also be limiting factors in the natural regeneration of this shrub. Additionally, clonal reproductive and clonal growth may be the most important ways for population expansion. However, as a pioneer species *C. mongolicum* is well adapted to propagate in mobile sand dune conditions and it is clear that it is unlikely to survive indefinitely in stabilized sand dune plantations.

## CONFLICT OF INTEREST

None declared.

## AUTHOR CONTRIBUTIONS

CMZ and BLF conceived and designed this study; SJG, JHZ, QLM, XJZ, WXZ, DJ, and QSY performed the field study; BLF analyzed the data and wrote the manuscript; ADM reviewed and supervised the manuscript; all authors contributed critically to the drafts and provided final approval for publication.
